# The Three-Cornered Alfalfa Hopper, *Spissistilus festinus*, Is a Vector of Grapevine Red Blotch Virus in Vineyards

**DOI:** 10.3390/v15040927

**Published:** 2023-04-07

**Authors:** Madison T. Flasco, Victoria Hoyle, Elizabeth J. Cieniewicz, Greg Loeb, Heather McLane, Keith Perry, Marc F. Fuchs

**Affiliations:** 1 School of Integrative Plant Science, Plant Pathology and Plant-Microbe Biology, Cornell University, Geneva, NY 14456, USA; 2 Plant and Environmental Sciences, Clemson University, Clemson, SC 29634, USA; 3 Department of Entomology, Cornell University, Geneva, NY 14456, USA; 4 School of Integrative Plant Science, Plant Pathology and Plant-Microbe Biology, Cornell University, Ithaca, NY 14853, USA

**Keywords:** *Grablovirus*, *Vitis vinifera*, *Spissistilus festinus*, *Geminiviridae*, transmission, acquisition

## Abstract

*Spissistilus festinus* (Hemiptera: Membracidae) transmit grapevine red blotch virus (GRBV, *Grablovirus*, *Geminiviridae*) in greenhouse settings; however, their role as a vector of GRBV in vineyards is unknown. Following controlled exposures of aviruliferous *S. festinus* for two weeks on infected, asymptomatic vines in a California vineyard in June and a 48 h gut clearing on alfalfa, a nonhost of GRBV, approximately half of the released insects tested positive for GRBV (45%, 46 of 102), including in the salivary glands of dissected individuals (11%, 3 of 27), indicating acquisition. Following controlled exposures of viruliferous *S. festinus* for two to six weeks on GRBV-negative vines in vineyards in California and New York in June, transmission of GRBV was detected when two *S. festinus* were restricted to a single leaf (3%, 2 of 62 in California; 10%, 5 of 50 in New York) but not with cohorts of 10–20 specimens on entire or half shoots. This work was consistent with greenhouse assays in which transmission was most successful with *S. festinus* exposed to a single leaf (42%, 5 of 12), but rarely occurred on half shoots (8%, 1 of 13), and never on entire shoots (0%, 0 of 18), documenting that the transmission of GRBV is facilitated through the feeding of fewer *S. festinus* on a restricted area of grapevine tissue. This work demonstrates *S. festinus* is a GRBV vector of epidemiological importance in vineyards.

## 1. Introduction

Red blotch disease was recognized as a serious threat to grape production beginning in the mid-2000s in California [1,2]. On red-berried wine grape cultivars, infected vines exhibit foliar reddening, whereas white-berried wine grape cultivars show chlorotic blotches and necrosis [2,3,4]. In addition to foliar symptoms, infected vines have delayed fruit ripening and reduced fruit quality, ultimately altering wine composition [3,4,5,6,7]. The disease is estimated to result in economic losses ranging from USD 2200 to 68,548 per hectare over the course of a vineyard’s productive lifespan [8].

Grapevine red blotch virus (GRBV) was found to be associated with diseased vines in the early 2010s [9,10] and later identified as the causal agent of the disease [11]. GRBV is a representative species of *Grapevine red blotch virus* of the genus *Grablovirus* in the family *Geminiviridae* [12,13]. Its single-stranded, circular DNA genome is composed of seven bi-directional open reading frames (ORFs). GRBV was recently speculated to have emerged from wild *Vitis* latent virus 1, a closely related geminivirus, about 9000 years ago [14]. Analyses of the genetic diversity of GRBV isolates revealed two statistically supported, distinct phylogenetic clades with up to 8.5% nucleotide sequence divergence [15]. Isolates of both clades are involved in red blotch disease etiology, though the biological significance, if any, of these two clades remains unknown [11].

*Vitis* spp. are the only known hosts of GRBV [3,16,17]. These include wine grape cultivars and rootstocks [2,3], table grapes [18], interspecific hybrids [19], Muscadine grapes [20], and free-living vines in Northern California [16,21,22] as well as in Southern Oregon [23].

GRBV is widespread in vineyards across the United States [15,24] and Canada [25,26,27]. Other countries have also reported GRBV, including South Korea [28], Switzerland [29], Mexico [30], Argentina [31], India [32], Italy [33], France [34], and most recently, Australia [35]. This widespread distribution of GRBV is attributed to the dissemination of infected planting material [2,3].

Despite the widespread distribution of GRBV throughout North America, secondary spread of GRBV has only been documented in Northern California [1,3,21,36] and Southern Oregon [23,37]. Spread of GRBV has not been documented in New York [36]. Spatiotemporal analyses of the distribution of GRBV in vineyards experiencing secondary spread implicated a flying hemipteran vector in the increased incidence of the virus [3,21,23,37]. The three-cornered alfalfa hopper (TCAH), *Spissistilus festinus* (Hemiptera: Membracidae), was identified early on as a vector candidate and shown to acquire and transmit GRBV in greenhouse settings [38,39,40]. Isolates of both GRBV phylogenetic clades are transmitted by *S. festinus* in the greenhouse [39]. The transmission mode is circulative and non-propagative with an extended acquisition access period (AAP) of 10 days [39,40]. The rate of the *S. festinus* mediated transmission of GRBV from infected to healthy grapevines in greenhouses is low (10–19%) [38,39,40].

No study has yet documented how *S. festinus* behave as a vector in vineyard settings. By analogy with greenhouse work [39,40], we hypothesized that *S. festinus* acquires GRBV from infected vines and transmits the virus to GRBV-negative vines in vineyards, contributing to the observed secondary spread. We further hypothesized that transmission in vineyards is inefficient, consistent with results from transmission assays conducted in the greenhouse [39,40]. In this study, we describe our work that confirms *S. festinus* as a GRBV vector of epidemiological relevance.

## 2. Materials and Methods

### 2.1. Plant Materials

Cuttings of healthy and GRBV-infected *V. vinifera* ‘Cabernet franc’ were collected during the winters of 2015 to 2018, as well as in 2020, and allowed to grow into mature plants in the greenhouse at 22 ± 3 °C with a 16 h:8 h light:dark photoperiod. Healthy and GRBV-infected grapevines were used as recipient and donor vines in transmission assays with *S. festinus*, respectively, as well as controls in diagnostic PCR. Some of these vines were monitored over one or two growing seasons following a dormancy period for at least 6 weeks in a cold room (4 °C) after lignified shoots were trimmed to 3–4 nodes.

Snap bean (*Phaseolus vulgaris*) ‘Hystyle’ was used as the donor plant material in transmission assays. Alfalfa (*Medicago sativa*) was used for gut clearing of *S. festinus* as it is a nonhost of GRBV [39]. Snap bean and alfalfa plants were used as rearing and feeding hosts for *S. festinus*. Both plant species were maintained in the greenhouse in controlled environmental chambers at 25 °C, a 16 h:8 h light:dark photoperiod, and 80% relative humidity.

Three vineyards were selected for this study. Two vineyards were in Napa County in California, and the third vineyard was in Ontario Country in New York. One of the California vineyards (2 ha) was planted in 2008 with ‘Cabernet franc’ (clones 214 and 623) vines grafted onto rootstock 101-14 Mgt (*V. riparia* × *V. rupestris*). The second California vineyard (1.5 ha) was planted in 2008 with ‘Cabernet Sauvignon’ (clones 4 and 169) grafted onto rootstock 101-14 Mgt. These two vineyards were chosen for this study because they have been the subjects of past and ongoing surveys for red blotch disease [3,36]. Because individual vines found in these blocks are monitored for disease symptoms and virus presence through PCR, the disease and virus status of vines for a given year are well-documented. The New York vineyard (0.1 ha) was planted in 2008 with ‘Cabernet franc’ (unknown clone) grafted onto 3309 Couderc (*V. riparia* × *V. rupestris*). GRBV is absent from the New York vineyard, as shown by repeated PCR testing of these vines and the absence of typical red blotch disease symptoms. In addition, no GRBV spread has been documented in the state [36].

### 2.2. Spissistilus festinus Population

The *S. festinus* used for this study were obtained from our laboratory-maintained colonies. Insects were first collected in October of 2015 from Yolo County in California and established on alfalfa in screened insect cages (BugDorm 6E610 Insect Rearing Cage, Taichung, Taiwan). Colonies were maintained in environmental chambers at 26 °C, with a 14 h:10 h light/dark photoperiod, and 85% relative humidity at Cornell AgriTech in Geneva, New York, as described by Flasco et al. (2021). The colony was supplemented with field-caught adults from Fresno County in California in November of 2015, as well as Yolo and San Joaquin Counties in California from 2016 to 2019, annually. Collected insects from all three counties were morphologically and genetically identical [41] and constitute our California-derived colony. Additional colonies were established on snap bean plants using individuals from the original alfalfa colony. 

### 2.3. GRBV Acquisition by S. festinus in the Vineyards

**Figure 1:**
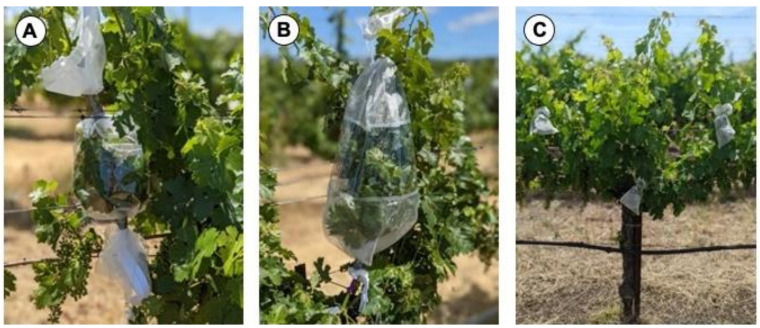
Distinct sleeve configurations used in transmission assays of grapevine red blotch virus by *Spissistilus festinus* in a ‘Cabernet Sauvignon’ vineyard. (**A**) A peppermint candy sleeve configuration allowing for cohorts of 10 *S. festinus* to feed on a restricted area of a shoot, (**B**) A tootsie roll sleeve configuration in which cohorts of 20 *S. festinus* were confined to an entire shoot, and (**C**) A lollipop sleeve configuration in which two *S. festinus* were allowed to feed on a single leaf on a middle–middle–bottom arrangement. Other lollipop sleeve configurations included middle–middle–top, top–middle–bottom, and middle–middle–middle.

To assess the possibility of *S. festinus* acquiring GRBV from infected ‘Cabernet franc’ vines in a California vineyard, aviruliferous adult insects were collected from our laboratory colonies at Cornell AgriTech and packaged in cohorts of 20 in 0.12 L deli containers (PlastiMade, Carteret, NJ, USA) with small holes punched in the lid and cup using a narrow screwdriver to ensure airflow. These containers were supplemented with alfalfa for *S. festinus* feeding to promote survival during shipment and were further confined in polypropylene containers with mesh snap lids (BugDorm, 960 mL, Taichung, Taiwan). These canisters were transported in a cardboard box that provided a third level of containment. In the vineyard, insect-rearing sleeves (BugDorm Insect Rearing Sleeve, L70 × W30 cm, Taichung, Taiwan) were secured to shoots with the bottom and top securely fastened to a shoot with duct tape. Afterwards, one deli container was delicately placed inside the sleeve, the zipper was fastened, and the lid of the deli cup was manually removed from the outside of the sleeve, allowing insects to feed on the grape tissue while preventing any escape into the external environment. Insects were placed in rearing sleeves on a portion of the shoot at either the middle or bottom canopy levels of known GRBV-infected grapevines. This sleeve configuration resembled that of a “peppermint candy” (Figure 1A). Aviruliferous insects were also placed on known GRBV-free vines in the same vineyards to serve as negative controls. Controlled-release experiments of *S. festinus* were in agreeance with USDA APHIS PPQ permits (P526P-20-04333 and P526P-21-02605).

After a two-week AAP, *S. festinus* were carefully collected using a D-cell-powered aspirator (Gemplers, Janesville, WI, USA) and placed in 960 mL polypropylene containers with snap lids containing live alfalfa plants collected from Yolo County, California. These insects were allowed to feed on alfalfa plants deemed free of field-obtained *S. festinus* based on visual inspections during the overnight shipment back to the laboratory in Geneva, New York to clear their mouthparts. After an additional 24 h of feeding, specimens were collected using a D-cell-powered aspirator and placed in a −20 °C freezer for later GRBV testing via PCR. Insects that remained alive at the time of the final collection underwent dissections, as described by Flasco et al. (2021), to ascertain the presence of GRBV in the salivary glands as an indication of virus acquisition. All leaves and petioles within the sleeves exposed to *S. festinus* were collected to confirm the presence of GRBV by PCR and determine the phylogenetic clade of the isolate.

### 2.4. Transmission Assays of GRBV by S. festinus from Infected Grapevines to Healthy Grapevines in the Greenhouse

Adult *S. festinus* were allowed to feed on GRBV-infected grapevines in the greenhouse for an AAP of 10 to 32 days in insect-rearing cages (BugDorm 2400F Insect Rearing Tent, Taichung, Taiwan). Then, cohorts of 12 or 15 insects were moved to caged (BugDorm 6E610 Insect Rearing Cage, Taichung, Taiwan) alfalfa plants, a nonhost of GRBV, for a 48 h gut clearing period [39]. Insects were moved to GRBV-free potted grapevines and confined using sleeves that were custom-made from cheesecloth in two configurations: (1) entire shoots or (2) smaller regions of a shoot. Insects were allowed to feed for a 4-day inoculation access period (IAP), after which insects were removed using a D-cell-powered aspirator for GRBV testing by PCR. Inoculated tissue was collected two weeks later for GRBV testing using PCR. Vines were subsequently maintained in greenhouses and monitored for symptom development; apical tissue was collected prior to and after one or two dormancy periods for GRBV testing by PCR. 

### 2.5. Inoculation of P. vulgaris with GRBV in the Greenhouse and Virus Acquisition by S. festinus

Two- to three-week old *P. vulgaris* plants received pinpricks along petioles in two cm increments through the use of a sterile dissecting needle dipped in a solid culture (Luria–Bertani medium supplemented with agar and kanamycin) of recombinant *Agrobacterium tumefaciens* C58C1 containing an infectious clone of GRBV isolate NY175 from phylogenetic clade 1 [11]. After a one-week incubation period, select inoculated petioles were tested for GRBV presence via PCR to ensure successful virus infection. The main stem of each plant remained untouched to avoid excess damage and stress to the plant. 

Because snap beans are a pseudo-systemic host of GRBV [39], the uninoculated main stem was wrapped in cheesecloth and secured with a twist-tie, concealing up to the first leaf node. Additionally, after the incubation period, all new, uninoculated tissue was pruned. These steps were taken to ensure that *S. festinus* were feeding almost exclusively on GRBV-infected tissue of inoculated plants to increase the opportunity for GRBV acquisition. *Spissistilus festinus* adults were exposed to the bean plants for a two-week AAP, a period that enables acquisition, as evidenced by the presence of GRBV in the salivary glands [39]. 

### 2.6. Agrobacterium tumefaciens Detection via PCR

The possible presence of recombinant *A. tumefaciens* C58C1 cells carrying the infectious GRBV clone NY175 was assessed by PCR in viruliferous *S. festinus* adults that acquired the virus by feeding on infected bean plants. These PCRs used primers designed in a fragment of *GlyA*, a gene from the *A. tumefaciens* C58C1 chromosomal background, encoding a serine hydroxymethyltransferase [42], and a HotStarTaq PCR kit (Qiagen, Germantown, MD, USA). The thermocycling conditions were as follows: 3 min at 95 °C, 30 cycles of 15 s at 95 °C, 15 s at 58 °C, and 30 s at 72, followed by 1 min at 72 °C. The PCR products were resolved by electrophoresis on agarose gels and visualized using UV illumination post-staining with GelRed (Biotium, Fremont, CA, USA). A 423 bp amplicon would indicate the presence of the recombinant bacteria, and no amplicon would indicate no detectable trace of the recombinant bacteria. DNA from inoculated *P. vulgaris* plants and DNA from *A. tumefaciens* containing the GRBV infectious clone that grew on selective solid media were used as positive controls in each PCR.

### 2.7. Transmission of Grapevine Red Blotch Virus by S. festinus from Infected P. vulgaris to Grapevines in the Vineyard

In June of 2020, after a two-week AAP on GRBV-infected *P. vulgaris*, viruliferous *S. festinus* were packaged in cohorts of 20 into 50 mL conical tubes with cheesecloth secured tightly using rubber bands or duct tape at the opening to ensure adequate airflow and proper containment. Conical tubes were placed in insect-rearing sleeves (BugDorm Insect Rearing Sleeve, L70 × W30 cm, Taichung, Taiwan) with the bottom securely fastened to the base of a shoot of a GRBV-free grapevine using duct tape in the ‘Cabernet franc’ vineyard in Geneva, New York (Ontario County). The top of the sleeve was secured to the shoot with duct tape, reminiscent of a “tootsie roll candy” (Figure 1B). Once the sleeve was secure, the cheesecloth was manually removed from the conical tube from the outside of the sleeve, allowing insects to feed on the grapevine tissue. This process was repeated for all of the vines exposed to *S. festinus* to ensure that the insects never had the opportunity to escape confinement. The insects were allowed to feed for a period of six weeks. The pyrethroid insecticide Mustang^®^ Maxx (active ingredient zeta-cypermethrin) was then applied after the six-week IAP by slightly opening the sleeves to delicately introduce the nozzle of the backpack sprayer and administer the contact insecticide to the shoots. After 24 h, the sleeves were removed, the dead insects aspirated, and three leaves exposed to the insects were collected. Subsequent tissue collections of leaves and petioles exposed to *S. festinus* occurred one, two, and three months post-insect removal. Apical tissue from a neighboring shoot (i.e., not exposed to *S. festinus*) of the inoculated vines was collected at the end of the growing season to assess potential GRBV movement from the site of inoculation. 

In June of 2021, the same experiment was repeated in the same ‘Cabernet franc’ vineyard in Geneva, New York on a new cohort of GRBV-free vines. In addition to the tootsie roll candy feeding configuration, viruliferous *S. festinus* were allowed to feed on smaller portions of the shoot in cohorts of ten. This configuration resembled that of a peppermint candy (Figure 1A). Adults were allowed to feed for a 2-week IAP to inoculate the healthy vines. As done previously, Mustang^®^ Maxx was applied prior to insect collection and sleeve removal. Inoculated leaves were allowed to remain on the vines until damage due to oviposition and insect-feeding-threatened leaf drop. In parallel, this experiment was repeated in the ‘Cabernet Sauvignon’ vineyard in Napa Valley in California in agreeance with USDA APHIS PPQ permits (P526P-20-04333 and P526P-21-02605). The overnight shipment of viruliferous *S. festinus* was performed with three containment levels, as described for the transfer of aviruliferous insects. In the vineyard, as was carried out previously, sleeves were secured to shoots prior to the removal of deli cup lids, preventing any escape. Insects were allowed to feed for up to seven weeks, a period longer than the lifespan of the insects on grapevine tissue, prior to sleeve removal. Inoculated and apical tissues were collected three months after sleeve removal. At the time of insect release, six basal leaves and petioles were collected to verify the GRBV-free status of each vine used in the study via PCR.

In June of 2022, transmission experiments were repeated in each vineyard on a new cohort of GRBV-free vines using exclusively a “lollipop” sleeve configuration (Figure 1C) in which two viruliferous *S. festinus* were placed on a single leaf using small insect-rearing bags (Bugdorm, L30 × W10 cm, Taichung, Taiwan). Basal leaves and petioles were collected as described above to ensure the virus status of each vine. After a two-week AAP, two insects were packaged in two-ounce deli cups (PlastiMade, Carteret, NJ, USA) supplemented with alfalfa shoots and with small holes added to allow for airflow. Five deli cups were placed securely in polypropylene containers with mesh snap lids for secure transport. In California, each inoculated vine received three lollipop sleeves at different canopy levels, with emphasis placed on the middle canopy (Figure 1C). Each inoculation site was on a different shoot of the vine. After the two-week IAP, the insects were carefully removed using a D-cell-powered aspirator. Inoculated leaves with oviposition or significant feeding damage were collected upon insect removal. The remaining leaves were collected three months after insect removal. Comparatively, vines inoculated in New York had four to five lollipop sleeves with a similar canopy focus. After the two-week IAP, Carbaryl 4L was applied to control insect pests in the New York vineyard, ensuring that all *S. festinus* were dead prior to collection. Insect removal was facilitated using a D-cell-powered aspirator and inoculated leaves with oviposition or significant feeding damage were collected. The New York vineyard block was monitored biweekly and inoculated leaves were collected over time, as senescence occurred due to feeding damage. 

Concurrently, the lollipop sleeve configuration was repeated in the greenhouse on potted, GRBV-free ‘Cabernet franc’ grapevines, and *S. festinus* were similarly allowed to feed for a two-week IAP. Each inoculated grapevine received a single lollipop sleeve. Greenhouse transmission experiments using the tootsie roll and peppermint candy sleeve configurations were similarly conducted using handmade sleeves of cheesecloth. Potted grapevines were placed in tented cages (BugDorm 2400F Insect Rearing Tent, Taichung, Taiwan) and insects were carefully removed using a D-cell-powered aspirator. Inoculated leaves remained on the vine but were removed prior to leaf drop due to feeding damage. 

All transmission experiments in the above work performed in New York and California in vineyards and greenhouses included aviruliferous *S. festinus* feeding on GRBV-negative grapevines as negative controls. 

### 2.8. Nucleic Acid Extraction from Plant and S. festinus Tissues

Genomic DNA was isolated from snap bean and grapevine material using an H.P. Plant DNA Mini Kit (Omega Bio-Tek, Norcross, GA, USA) or a MagMAX-96 Al/ND Isolation Kit (Thermo Fisher Scientific, Waltham, MA, USA) on a KingFisher instrument. Genomic DNA was isolated from whole *S. festinus* bodies or dissected *S. festinus* organs using an E.Z.N.A. Insect DNA kit (Omega Bio-Tek) or a MagMAX-96 Al/ND Multi-Sample Ultra Kit (Thermo Fisher Scientific, Waltham, MA, USA) on a KingFisher instrument. 

### 2.9. GRBV Detection via PCR and qPCR

The presence of GRBV was determined in plant tissue and *S. festinus* samples via multiplex PCR using genomic DNA and primer pairs hybridizing to the coat protein and replication ORFs of the viral genome [3,15,37]. DNA amplicons were analyzed by gel electrophoresis and visualized using UV illumination post-staining with GelRed (Biotium, Fremont, CA, USA).

The relative quantification of GRBV in *V. vinifera* samples was assessed using SYBR Green reagents (iTaq Universal SYBR Green Supermix, Bio-Rad, Hercules, CA, USA) with the primers pREP3v and pREP4v designed in the ORF C1 coding RepA, as well as a primer pair designed in the plant nicotinamide adenine dinucleotide phosphate gene [43]. Negative controls included sterile water and nucleic acids extracted from noninfected *V. vinifera*. GRBV testing via qPCR in *S. festinus* used the above-mentioned pREP3v and pREP4v primer pair [43] in addition to the *S. festinus* primers Sf18SFor (5′-GTGAGGTCTTCGGACTGGTG-3′) and Sf18Srev (5′- GGTTCACCTACGGAAACCTTG-3′). Negative controls included sterile water and nucleic acids from colony-maintained insects. Each sample was run in triplicate on a Bio-Rad C1000 Touch Thermocycler. GRBV was quantified using the relative ∆∆Ct method to calculate the fold difference of GRBV DNA between two samples.

### 2.10. GRBV Phylogenetic Clade Determination via Restriction Digestion of Rep PCR Amplicons

To determine the phylogenetic clade of GRBV isolates in plant samples and *S. festinus* specimens, PCR products amplifying a fragment of the GRBV replication ORF [3,15,37] from plant and insect nucleic acids were restriction digested with AleI-v2 (New England Biolabs, Ipswich, MA, USA) at 37 °C for two hours. Each reaction contained 5 µL of PCR reaction and 2 µL of rCutSmart™ buffer (New England Biolabs), for a final volume of 20 µL. Digestions were resolved by electrophoresis on agarose gels and visualized using UV illumination post-staining with GelRed (Biotium, Fremont, CA, USA). In addition, PCR products were Sanger sequenced at the Cornell Biotechnology Resource Center in Ithaca, New York, to determine the accuracy of the restriction digests in distinguishing GRBV isolates from distinct phylogenetic clades. GRBV sequences were assembled using the DNASTAR Lasergene software suite, version 14.1.

### 2.11. Statistics

Statistical analyses were performed on the titer of GRBV in infected grapevines and viruliferous insects according to the calculated expression fold change values ($2^{-\Delta \Delta \mathrm{Ct}}$) as determined via qPCR. Analyses of variance (ANOVA) and Welch’s *t*-tests were performed in the RStudio program (the R Project for Statistical Computing). The significance level was set at α = 0.05.

## 3. Results

### 3.1. GRBV Titer Is Consistent in the Middle and Lower Canopy of Infected ‘Cabernet franc’ Vines Selected for Acquisition Experiments in the Vineyard in June

Acquisition experiments of GRBV by *S. festinus* adults were performed in a diseased ‘Cabernet franc’ vineyard in California in June 2022, a seasonal period during which no red blotch disease symptoms are apparent. Twelve vines were selected for this work, 10 were infected with GRBV and two that were noninfected, as determined by multiplex PCR in 2020 and 2021. 

**Figure 2:**
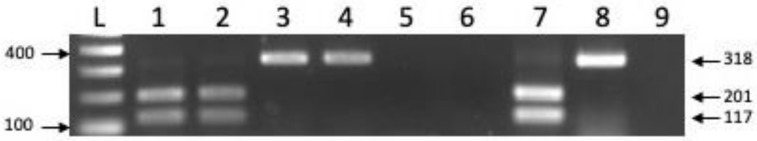
Phylogenetic clade differentiation of grapevine red blotch virus (GRBV) isolates via restriction digestion. PCR amplicons of the GRBV replication ORF were digested by AleI-v2 and resolved by agarose gel electrophoresis and GelRed staining. Amplicons obtained from the vines infected with a GRBV clade 1 isolate were cut into two bands, approximately 201 and 117 bp in length, while those obtained from the vines infected with a GRBV clade 2 isolate remained uncut at 318 bp in length. Lanes 1 and 2, GRBV clade 1-infected grapevines from a California vineyard; lanes 3 and 4, GRBV clade 2-infected grapevines from a California vineyard; lane 5, a healthy grapevine from a California vineyard; lane 6, no template control; lane 7, a GRBV clade 1-infected potted grapevine in a greenhouse following *Agrobacterium tumefaciens*-mediated inoculation; and lane 8, a GRBV clade 2-infected potted grapevine in a greenhouse following *A. tumefaciens*-mediated inoculation. Lane L: 100 bp molecular weight marker.

A simple restriction digest assay of the 318 bp Rep PCR amplicons obtained from GRBV-infected vines was developed to distinguish GRBV isolates from distinct phylogenetic clades. PCR amplicons digested with AleI-v2 produced two fragments of approximately 201 and 117 bp in length for GRBV clade 1 isolates, while a single uncut 318 bp fragment was obtained for GRBV clade 2 isolates (Figure 2). The restriction digest results were consistent with the previous characterization of the GRBV isolates in the 10 infected vines in 2011 and again in 2020, showing isolates of phylogenetic clade 1 in five vines and isolates of phylogenetic clade 2 in the other five vines. Additionally, the typical leaf reddening characteristic of red blotch disease was confirmed in October 2020 and 2021 in the 10 GRBV-infected vines but not in the two noninfected vines. 

*S. festinus* cohorts fed on the middle canopy of seven vines and the lower canopy of five vines in a peppermint candy sleeve configuration in June. After a two-week AAP, all the grapevine tissues fed upon by *S. festinus* were collected to confirm the presence of GRBV and assess virus titer. The previously symptomatic and infected vines (100%, 10 of 10) tested positive for GRBV by multiplex PCR, while the two negative control vines did not (0%, 0 of 2), as expected. A comparative analysis of the GRBV titer in the 10 infected vines using the calculated $2^{-\Delta \Delta \mathrm{Ct}}$ indicated no significant differences between the middle (*n* = 6) and lower (*n* = 4) canopy levels (*p* = 0.577). Similarly, there were no differences in terms of the virus titers between vines infected with GRBV isolates from phylogenetic clade 1 (*n* = 5) and clade 2 (*n* = 5) (*p* = 0.921), or by clade per canopy position (*p* = 0.958). These results indicated that *S. festinus* were exposed to similar virus titer in the GRBV-infected vines selected for the acquisition experiments in the vineyards.

### 3.2. Spissistilus festinus Acquires GRBV from Infected Grapevines in the Vineyards

After a two-week AAP on previously selected GRBV-infected grapevines in a ‘Cabernet franc’ vineyard in California, all 240 *S. festinus*, both alive and dead, were accounted for and recovered at the completion of the controlled release experiment. Following shipment on alfalfa plants to the laboratory in Geneva, New York, a total of 124 insects were unable to be tested as they were crushed during transport. Of the adult insects that were recovered and fed for 24 h on alfalfa in a greenhouse, close to half (45%, 46 of 102) tested positive for GRBV by multiplex PCR. All 14 of the *S. festinus* adults recovered from noninfected grapevines tested negative for GRBV via multiplex PCR, as expected.

Following the dissections of 27 individual *S. festinus* and testing for GRBV using multiplex PCR, 11% (3 of 27) of heads as well as salivary glands and 67% (18 of 27) of guts were positive. Of the three head and salivary gland samples that were positive for GRBV, two had a virus isolate of phylogenetic clade 1 and one had an isolate from phylogenetic clade 2, as determined by a restriction digest of the PCR product, indicating the acquisition of virus isolates from both phylogenetic clades in the vineyard. Similarly, of those gut tissues that tested positive for GRBV, 10 contained a phylogenetic clade 1 isolate (10 of 18) and eight contained a clade 2 isolate (8 of 18). The phylogenetic clade identified in the head and gut tissues of the insects corresponded to the phylogenetic clade present in the vine upon which they were feeding, as expected. Three additional dissections were conducted on individuals that fed on GRBV-free grapevines, all of which tested negative for GRBV via PCR in both the head and the gut. The detection of GRBV in the head and salivary glands after a gut clearing period on alfalfa indicated the acquisition of GRBV by *S. festinus* upon feeding on infected, asymptomatic grapevines in the vineyard in June. 

### 3.3. GRBV Titer in S. festinus after Acquisition from Infected Grapevines in the Vineyard Vary by Insect Tissue Type

**Figure 3:**
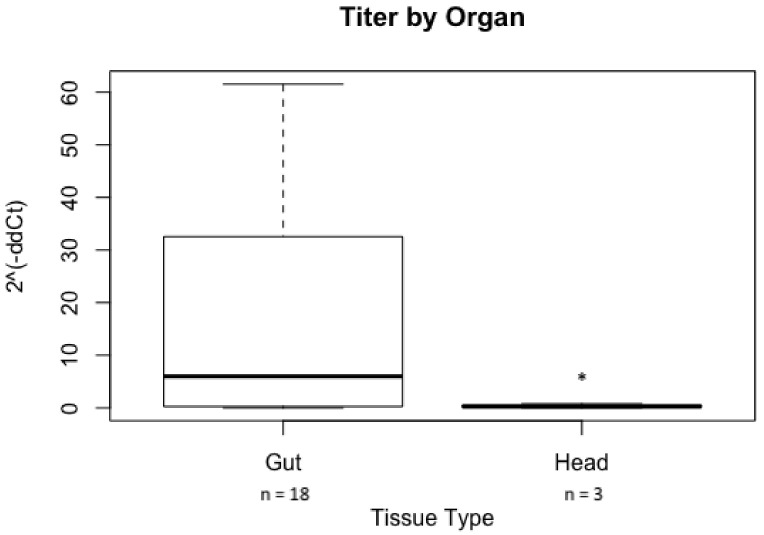
Titer of grapevine red blotch virus (GRBV) in *Spissistilus festinus* tissue determined by qPCR. Dissected head as well as salivary glands (Head) and gut (Gut) from *S. festinus* exposed to GRBV-infected *Vitis vinifera* ‘Cabernet franc’ for two weeks in a California vineyard followed by 48 h on alfalfa were tested. The number of individuals dissected and tested is shown. The error bars indicate the standard error of the mean. The star above the standard error bar indicates significant differences between the ΔΔCt expression fold change values ($2^{-\Delta \Delta \mathrm{Ct}}$), as determined by Welch’s *t*-test (*p* < 0.05).

A significant difference in GRBV titer was found in *S. festinus* tissue after acquisition with less virus in the head as well as the salivary glands (*n* = 3) versus the gut (*n* = 18), as shown by a statistical analysis of the calculated $2^{-\Delta \Delta \mathrm{Ct}}$ (*p* = 0.019) (Figure 3). In addition, no significant difference in virus titer was found when comparing *S. festinus* gut tissues following feeding on different canopy levels (*p* = 0.577) and vines infected by GRBV isolates from a different phylogenetic clade (*p* = 0.577). These results indicated an inefficient virus movement from the gut to the salivary glands of *S. festinus* for acquisition.

### 3.4. The Rate of GRBV Ingestion by Spissistilus festinus from Infected Grapevines in the Vineyard Is Similar between Phylogenetic Clades and Canopy Position

When dissections of *S. festinus* individuals were not possible, whole bodies were individually tested for GRBV as a measure of GRBV ingestion. A total of 75 whole *S. festinus* bodies collected from GRBV-infected vines were tested by multiplex PCR, of which 28 tested positive (37%). These findings were confirmed via qPCR. In addition, a comparative analysis of the ΔΔCt expression fold change ($2^{-\Delta \Delta \mathrm{Ct}}$) indicated no significant difference in the ingestion of GRBV by *S. festinus* based on the phylogenetic clade (*p* = 0.289), canopy position (*p* = 0.495), or virus titer by clade per canopy position (*p* = 0.538). These data showed that *S. festinus* is capable of ingesting GRBV at similar rates regardless of the virus phylogenetic clade and the vine canopy level on which they were feeding.

### 3.5. Spissistilus festinus Transmit GRBV from Infected Grapevines to Healthy Grapevines in the Greenhouse

For virus transmission experiments in the greenhouse, *S. festinus* were exposed to GRBV clade 2-infected potted grapevines for an AAP of 10 to 32 days, followed by a 48 h gut clearing period on alfalfa. Then, cohorts of 15 insects were placed along an entire shoot of a GRBV-free potted grapevine and allowed to feed for a four-day IAP. Similarly, cohorts of 12 insects were placed on restricted areas of a shoot and allowed to feed for a four-day IAP. Inoculated tissue was collected two weeks after insect removal and tested for GRBV by multiplex PCR and qPCR. When feeding on the whole shoot during the IAP, no inoculated leaves tested positive (0%, 0 of 9) while the inoculated shoot of one vine tested positive when feeding on the restricted area (8%, 1 of 13). Despite little to no transmission, most insects tested positive after feeding on the whole shoot (92%, 124 of 135) or restricted shoot area (92%, 102 of 111). Negative control vines (0%, 0 of 6) and aviruliferous insects (0%, 0 of 68) tested negative for GRBV, as expected. These results indicated that *S. festinus* is capable of transmitting GRBV from infected to healthy grapevines in the greenhouse, albeit at a low efficiency.

In parallel, *S. festinus* were allowed to feed on GRBV-inoculated bean plants for two weeks to acquire GRBV. Insects were then placed in cohorts of two onto a single leaf of a potted GRBV-free ‘Cabernet franc’ grapevine and allowed to feed for two weeks in the greenhouse. Upon insect and sleeve removal, inoculated leaves were sampled based on leaf health. Subsequent testing resulted in 42% (5 of 12) of inoculated leaves testing positive for GRBV via multiplex PCR. Transmission did not occur when utilizing the tootsie roll (0%, 0 of 4) or peppermint candy (0%, 0 of 5) sleeve configurations. All of the negative control vines tested negative for GRBV (0%, 0 of 4), as expected. These data showed that the transmission of GRBV by *S. festinus* is more efficient when the feeding area is restricted on greenhouse-grown grapevines and few insects are used.

### 3.6. Restricting the Feeding Area of S. festinus on Grapevine Tissue and Limiting the Number of Insects Increase the Ability to Document GRBV Transmission in the Vineyard

Controlled release experiments were conducted using viruliferous *S. festinus* in two vineyards utilizing an iterative approach that restricted the area of feeding and limited the number of insects. Prior to the controlled release of viruliferous *S. festinus*, it was important to test whether insects feeding on GRBV-infected beans could acquire *A. tumefaciens* C58C1 carrying the infectious GRBV clone NY175. Inoculated bean plants contained detectable recombinant *A. tumefaciens* DNA in the petiole at the site of inoculation and in the two cm area between pinpricks (100%, 6 out of 6), but no *A. tumefaciens* DNA was detectable in leaf tissue beyond the agroinoculation sites (0%, 0 of 2). All of the bean tissue tested positive for GRBV (100%, 8 of 8), as expected. No detectable *Agrobacterium* DNA was present (0%, 0 of 6) in any of the *S. festinus* tested two weeks post-feeding on GRBV-infected bean tissue, as shown by PCR, despite testing positive for GRBV (100%, 6 of 6). These results showed no detectable carry over recombinant *A. tumefaciens* in *S. festinus* following feeding on GRBV-infected bean plants.

When insects were allowed to feed on entire shoots using the tootsie roll sleeve configuration, no transmission was obtained in either the New York vineyard (0%, 0 of 14) or the California vineyard (0%, 0 of 6) (Table 1). This pattern continued with the peppermint candy sleeve configuration in which no vines tested positive (0%, 0 of 11 in New York and 0%, 0 of 15 in California) in the year that they were inoculated (Table 1). 

Preliminary lollipop sleeve configuration work in 2021 situated single inoculation sites at the base of the canopy of five vines in the New York vineyard, none of which tested positive for GRBV (0%, 0 of 5) by PCR; however, the repeated use of the lollipop sleeve configuration in New York and California in June of 2022, including additional sites within the canopy, yielded successful transmission (Table 1). In New York, close to half of the inoculated vines (45%, 5 of 11) tested positive for GRBV using multiplex PCR (Table 1). Each of the vines in which transmission occurred had tissue from a single lollipop sleeve test positive for GRBV via multiplex PCR in early October with three lollipop sleeves located in the middle canopy and two in the lower canopy. Inoculated leaves for this experiment were collected just prior to leaf drop based on senescence. In California, grapevine tissue from one lollipop sleeve located in the middle canopy of a single vine tested positive for GRBV by multiplex PCR (5%, 1 of 20) (Table 1) following direct leaf collection after insect removal (in June 2022). Grapevine tissue from an additional lollipop sleeve collected in the upper canopy of the same vine in October also tested positive for GRBV via multiplex PCR. Taken together, grapevine tissue in 10% (5 of 50) of the lollipop sleeves in the New York vineyard and 3% (2 of 62) of the lollipop sleeves in the California vineyard tested positive for GRBV via PCR in 2021–2022. These results indicated that restricting the area of potential GRBV inoculation, as well as limiting the number of insects feeding, increased the transmission of GRBV by *S. festinus* in the vineyards.

### 3.7. GRBV Is Detected in S. festinus-Inoculated Grapevines after One Dormancy, but No Disease Symptoms Are Apparent

Grapevine shoot tissue exposed to viruliferous *S. festinus* from previous years’ studies was collected from vines in the New York and California vineyards and tested for GRBV via multiplex PCR. No vines exposed to viruliferous *S. festinus* using the tootsie roll sleeve configuration tested positive for GRBV in either 2021 (0%, 0 of 10) or 2022 (0%, 0 of 14) in the New York vineyard following one dormancy. Conversely, two vines (18%, 2 of 11) inoculated by viruliferous *S. festinus* in 2021 via the peppermint candy sleeve configuration tested positive for GRBV by PCR in 2022 in the New York vineyard. Neither of these two vines exhibited foliar GRBV symptoms. Similarly, no tissue of vines exposed to viruliferous *S. festinus* in 2021 from the tootsie roll (0%, 0 of 6) or the peppermint candy (0%, 0 of 15) sleeve configurations tested positive for GRBV in 2022 in the California vineyard, and none of these vines exhibited foliar red blotch symptoms. In both the New York and California vineyards, tissue collected from the neighboring non-inoculated shoots of vines exposed to viruliferous *S. festinus* tested negative for GRBV by PCR in the year of inoculation (2020: 0%, 0 of 10; 2021: 0%, 0 of 36), the following year (2021: 0%, 0 of 10; 2022: 0%, 0 of 36), and two years following inoculation (0%, 0 of 10). These data showed that GRBV transmission may not be detectable beyond the initial inoculated tissue until at least one year after initial exposure to viruliferous *S. festinus*. In addition, more than one year may be needed for systemic infection to occur and for disease symptoms to develop following the *S. festinus* mediated inoculation of GRBV in the vineyards.

### 3.8. Co-Infection of GRBV Isolates from Both Phylogenetic Clades Can Be Detected in a Single Vine in the Vineyard

Since the natural spread of GRBV is reported in the ‘Cabernet Sauvignon’ vineyard used in this study to document *S. festinus* mediated transmission of GRBV [1,3,36], it was essential to determine the genetic makeup of the GRBV isolates found in infected vines that viruliferous *S. festinus* carrying a GRBV clade 1 isolate were exposed to in 2022. This was critical to ascertain that no natural *S. festinus*-mediated transmission of GRBV had occurred in the selected vines prior to 2022, potentially interfering with any transmission work performed in the California vineyard. 

The restriction digest assay of Rep amplicons obtained by PCR was applied to the GRBV isolates detected in the single vine that tested positive for GRBV via PCR following exposure to viruliferous *S. festinus* on a single leaf at the bottom, middle, and upper canopy levels using the lollipop sleeve configuration in the California vineyard. The results indicated that the basal leaves of this vine, as well as the lower canopy inoculated leaf, were infected with a GRBV clade 2 isolate, while the middle canopy inoculated leaf was found to be infected with a GRBV clade 1 isolate, the same phylogenetic clade carried by viruliferous *S. festinus* upon the collection in June. Collection in October of the inoculated leaf in the upper canopy of the same vine indicated the presence of a GRBV clade 1 isolate. These results confirmed the successful transmission of GRBV in this vine utilizing controlled *S. festinus* inoculation in the middle and upper canopies, but also suggested infection with a GRBV clade 2 isolate in the lower canopy, likely resulting from natural *S. festinus*-mediated transmission prior to the controlled release of viruliferous *S. festinus*. The presence of GRBV isolates from both phylogenetic clades in a single vine shows the potential for co-infection in the vineyard.

### 3.9. Girdling and Foliar Reddening Due to S. festinus Feeding in the Vineyard Is Not Indicative of GRBV Transmission

**Figure 4:**
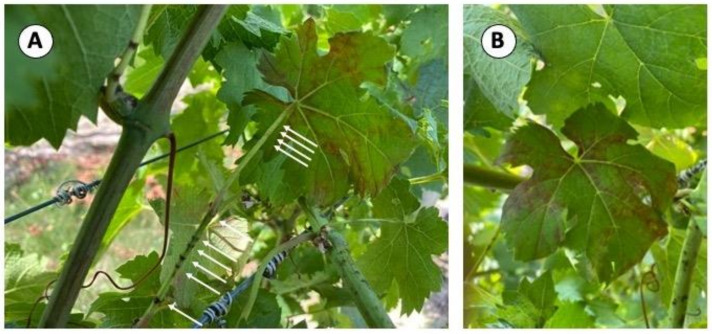
Girdles and foliar reddening observed after *Spissistilus festinus* feeding on ‘Cabernet franc’ grapevine tissue in New York. Utilizing the tootsie roll sleeve configuration, 20 viruliferous *S. festinus* were allowed to feed on the length of a shoot. Upon the removal of the insects, the number of girdles was observed, and foliar reddening was monitored for the remainder of the growing season. (**A**) Ten girdles indicated by the white arrows due to *S. festinus* feeding and reddening on the abaxial surface of the leaf resembling red blotch disease symptoms. (**B**) Foliar reddening observed on the adaxial surface of the leaf due to the 10 girdles resembling grapevine red blotch symptoms. Despite the observed reddening upon feeding by viruliferous *S. festinus*, GRBV was not present in the inoculated leaf when tested via multiplex PCR.

After the two-week IAP with viruliferous *S. festinus* release experiment in New York in 2021, the number of girdles due to feeding damage on shoots and petioles within the sleeve (Figure 4A) was recorded (Table 2). Additionally, over the course of three months, foliar reddening (Figure 4B) reminiscent of GRBV symptomology, was recorded (Table 2). Girdling was observed on all of the lollipop-sleeved vines (100%, 5 of 5), with an average of 2.2 girdles per inoculated sleeve, but foliar reddening was not apparent (0%, 0 of 5). Similarly, girdling was observed on all of the peppermint candy-sleeved vines (100%, 11 of 11), averaging 14.3 girdles per sleeve. Red leaves were seen on almost all of the inoculated vines (91%, 10 of 11) with an average of 2.1 red leaves per sleeve (Table 2). Those vines exposed to viruliferous *S. festinus* in the tootsie roll sleeve configuration showed girdling (100%, 4 of 4), with an average of 22 girdles per sleeve (Table 2). Foliar reddening was observed on all of the vines (100%, 4 of 4), averaging six red leaves per sleeve. The vines exposed to aviruliferous *S. festinus* utilizing the peppermint candy and tootsie roll sleeve configurations exhibited 15 girdles with 3 red leaves and 25 girdles with 4 red leaves, respectively. Despite clear evidence of *S. festinus* feeding and subsequent leaf reddening, GRBV was not detected on any of the inoculated shoots (0%, 0 of 20). These results showed that girdling and foliar reddening due to *S. festinus* feeding damage is independent of GRBV inoculation and multiplication in grapevine tissue. Furthermore, they revealed that feeding damage by *S. festinus* was less severe in the lollipop compared with the peppermint candy and tootsie roll sleeve configurations.

## 4. Discussion

In this work we definitively showed that *S. festinus* is a vector of GRBV in vineyard settings. Using the extended AAP previously described in greenhouse experiments [39,40], we showed, for the first time, acquisition of GRBV by *S. festinus* after a two-week feeding period on infected grapevines in the vineyard. The high number of *S. festinus* individuals testing positive for GRBV (45%, 46 out of 102) supported feeding upon infected vines in vineyards, as previously reported [1,17]. More importantly, insect dissections after gut clearing on alfalfa indicated the presence of GRBV in the head and salivary glands of some of the individuals tested (11%, 3 of 27), consistent with the circulative mode of GRBV transmission previously documented [39]. Nonetheless, lower concentrations of GRBV DNA were found in the head and salivary glands post-acquisition compared to the guts (Figure 3). A differential amount of GRBV in various *S. festinus* tissues was similarly described in greenhouse settings [39]. These consistent findings highlight the need for studies to understand the barriers to virus movement within an insect. Taken together, this work solidifies the necessity to distinguish GRBV ingestion through feeding on an infected grapevine and acquisition via the circulative movement of the virus within an insect body to reach the salivary glands, a necessary step for transmission.

Influencing *S. festinus*-mediated GRBV transmission experiments in the vineyard were preliminary transmission assays in the greenhouse that indicated promising results when the area of *S. festinus* feeding was restricted and the number of insects used during the IAP was limited. Indeed, a higher rate of transmission was achieved when two *S. festinus* were restricted to a single leaf using the lollipop sleeve configuration (42%, 5 of 12), compared with a lower transmission rate when 12 *S. festinus* were restricted on portions of shoots using the peppermint candy (6%, 1 of 18) and 20 *S. festinus* using the tootsie roll sleeve configurations (0%, 0 of 22).

Viruliferous *S. festinus* releases in the vineyard were conducted in an iterative approach, building upon each growing season. In 2020, conical tubes were used for the transporting and releasing of insects. These proved to be detrimental when *S. festinus* would remain in the tubes and drown after rain events. Utilizing deli cups supplemented with holes to allow for airflow in 2021 and 2022 proved to be key in subsequent releases. No transmission was evident in 2020, during which insects were exposed to an entire shoot. This pattern persisted through 2021 when insects were restricted to a smaller feeding area. It was not until single leaf inoculations occurred with only two insects in the lollipop sleeve configuration that GRBV was detectable (17%, 6 of 36) the same year of inoculation (Table 1). No transmission was documented for the year of GRBV inoculation in the vineyard using the peppermint candy and tootsie roll sleeve configurations with more viruliferous *S. festinus* restricted on entire shoots or half shoots (Table 1).

Interestingly, extensive foliar reddening, likely due to anthocyanin accumulation, was observed on leaves as a result of insect feeding, particularly with the peppermint candy and tootsie roll sleeve configurations, though none of the seemingly symptomatic leaves proved to be infected with GRBV (Table 2). Limited girdling and no foliar reddening occurred upon *S. festinus* feeding on lollipop-sleeved grapevine tissue in comparison with the other two sleeve configurations (Table 2). This reduced feeding damage indicated less physically injured phloem tissue in lollipop-sleeved leaves and may explain why GRBV was preferentially able to replicate and eventually move from the inoculate site in this sleeve configuration, resulting in higher detectable transmission rates. This observation is supported by the likelihood of the phloem restriction of GRBV, an assumption made by analogy with most of the other viruses in the *Geminiviridae* family [44,45].

Due to the excess girdling caused by *S. festinus* feeding [16,38,46,47,48,49], balancing adequate feeding to encourage virus transmission while mitigating damage that would altogether halt virus infection was crucial. Because *S. festinus* use grapevines as opportunistic hosts and recorded specimens in vineyards are relatively low [3,38,46,47,48,49], we hypothesize that this insect vector visits vineyards transiently and does not interact with any one vine for an extended period of time. This is unlikely to result in the extensive feeding damage observed in our controlled settings (Table 2). Less feeding damage in uncontrolled vineyard conditions likely favors virus transmission, as documented here. Furthermore, the removal of inoculated leaves for virus testing may prevent systemic virus infection, despite successful transmission to an individual petiole. These risks were alleviated by reducing the IAP from six to two weeks, and only collecting leaves just prior to drop due to senescence. In fact, these conditions provided the first cases of successful *S. festinus* mediated transmission of GRBV to healthy grapevines. As a result, two vines inoculated via the peppermint candy sleeve configuration (18%, 2 of 11) tested positive for the first time a year after exposure to viruliferous *S. festinus* in the New York vineyard. Since these vines did not exhibit symptoms of GRBV despite positive PCR results, we hypothesize that GRBV may not show disease symptoms until at least one to two years post-inoculation by viruliferous insects. Across our experiments in the New York and California vineyards, GRBV was detected after two weeks of viruliferous *S. festinus* feeding, four months after feeding, or even a year later. An extended 2- to 64-week latency period (the time between inoculation and the detection of the virus) for GRBV is unique for a grapevine pathogen. For grapevine leafroll-associated virus 3 (GLRaV 3), the virus was detectable in inoculated vines three months after mealybug-mediated transmission, and infected vines became symptomatic the following year [50]. Pierce’s disease results in symptoms appearing within the same year of feeding by the blue green sharpshooter [51].

Populations of *S. festinus* in vineyard settings peak in late June and early July in Northern California [3,48]. It proved crucial to conduct experiments in both New York and California to reflect the presence of *S. festinus* in the vine canopy rather than performing experiments at peak symptom development and highest virus titer in infected vines in September/October [2,3]. Consequently, we were able to document the acquisition and transmission of GRBV by *S. festinus* in June. Interestingly, when considering the successful lollipop sleeve configuration for transmission experiments, one leaf tested positive from the upper canopy (one out of seven), three from the middle (three out of seven), and three from the lower canopy (three out of seven) in the vineyards. This generally reflects observed feeding damage in California vineyards, which occurs primarily in the middle canopy (MTF, unpublished observations). The successful acquisition and transmission of GRBV by *S. festinus* in June highlights the role of asymptomatic infections of GRBV in the vineyard. Despite not showing symptoms and having lower virus titer in June when compared to later in the growing season [43], these asymptomatic vines allowed for virus acquisition and transmission by *S. festinus*.

A challenge to this work was the logistical difficulties in carrying out concurrent experiments in vineyards in both New York and California. Thousands of *S. festinus* needed to be reared for the controlled-release experiments that studied acquisition in California as well as transmission in both New York and California. Shipping live *S. festinus* from New York to California, and back again, necessitated creative approaches to facilitate the insects’ transition from growth chambers with controlled environmental conditions to the natural elements of vineyard settings. Despite precautions, stress on the insects due to the shipments and the need to adjust to a new climate likely contributed to the lower rate of transmission in the California vineyard (2%, 1 of 41) compared with the New York vineyard (12%, 5 of 41). In addition, while all of the insects released in sleeves were recovered in the California vineyard, many were crushed within the heavy clay soil that the alfalfa plants were rooted in during the return shipment and were unable to be tested. Furthermore, the documented spread of GRBV in the California vineyards selected for this study [1,3,37] required the development of a simple, fast, and accurate diagnostic assay for the genotyping of virus isolates to identify possible interfering isolates, particularly those that might influence controlled transmission assays due to the natural spread of GRBV by *S. festinus*. We picked a restriction digest assay of GRBV Rep amplicons rather than a more time-consuming sequence analysis of a fragment spanning the long intergenic region of GRBV [37] or Rep fragments [15] as previously done, although both methodologies were equally effective at genotyping virus isolates. The characterization of GRBV isolates via PCR followed by restriction digests, was essential for reaching solid conclusions on the occurrence of the *S. festinus*-mediated transmission of GRBV in the ‘Cabernet Sauvignon’ vineyard in California.

Higher rates of GRBV transmission occurred in the greenhouse than in the vineyard for each sleeve configuration. Overall, 14% (6 of 43) of potted vines exposed to viruliferous *S. festinus* in the greenhouses tested positive for GRBV by PCR, while only 7% (6 of 82) of the grapevines exposed to viruliferous *S. festinus* in the vineyards tested positive for GRBV by PCR. This result was not unexpected, as similar observations have been made for other grapevine pathogens, such as GLRaV 3 [50] and *Xylella fastidiosa* [52]. A similar pattern is seen outside of grapevine pathogens, with higher rates of transmission in greenhouses than in fields for banana bunchy top virus [53].

This study is the first to document the coinfection of a clade 1 isolate and a clade 2 isolate of GRBV in the same vine. Tissue that was collected in June indicated that the lower canopy was infected with a clade 2 isolate, while the controlled release of viruliferous *S. festinus* resulted in the presence of a GRBV clade 1 isolate in the middle (two weeks post-inoculation) and upper leaves (four months post-inoculation). The presence of GRBV clade 1 in the upper leaf could be due to *S. festinus* inoculation or virus translocation from the infected middle leaf. Additional work is necessary to understand the movement of GRBV within a vine from its inoculation site. Similarly, more work is needed to assess the effects of the coinfection of isolates of both phylogenetic clades, as seen with GLRaVs [54,55,56], or the potential for competitive exclusion between GRBV isolates of the two phylogenetic clades.

*Spissistilus festinus* is a vector of GRBV in the vineyard, but other vectors may exist, as other arthropod vector candidates of the Membracidae and Cicadellidae families have been reported [3,17,36,57,58]. While found in diseased vineyards, none of these vector candidates have yet been shown to acquire GRBV in the greenhouse or in the vineyard.

In summary, we documented *S. festinus* acquiring GRBV from infected vines and transmitting GRBV to healthy vines in the vineyard, solidifying its role as an epidemiological vector of red blotch disease. This work highlighted the need to balance adequate feeding time for virus transmission while minimizing feeding damage, a feat successful when two *S. festinus* individuals were restricted to a single grapevine leaf. Striking this balance showed that foliar reddening due to insect feeding is not indicative of GRBV transmission. Our work in the vineyards is consistent with work done in the greenhouse. This research can serve as a basis for advancing our understanding of disease epidemiology and for studying virus–vector–host relationships in the vineyard.

## Figures and Tables

**Table 1:** Transmission of grapevine red blotch virus (GRBV) to grapevines in a New York and a California vineyard by *Spissistilus festinus* using various sleeve configurations for inoculation after a two-week acquisition period on infected *Phaseolus vulgaris* plants.

Sleeve Configuration	2020 ^a^		2021 ^b^		2022 ^c^		Total ^d^		Total ^f^
	NY ^e^	CA	NY	CA	NY	CA	NY	CA	
Tootsie roll ^g^	0/10	n/a	0/4	0/6	n/a	n/a	0/14	0/6	0/20
Peppermint candy ^h^	n/a	n/a	0/11	0/15	n/a	n/a	0/11	0/15	0/26
Lollipop ^i^	n/a	n/a	0/5	n/a	5/11	1/20	5/16	1/20	6/36
Total ^j^	0/10		0/41		6/31		6/82		

^a^ Viruliferous *S. festinus* were allowed to feed for six weeks on healthy grapevines. ^b^ Viruliferous *S. festinus* were allowed to feed for two weeks on healthy grapevines in New York and up to seven weeks in California. ^c^ Viruliferous *S. festinus* were allowed to feed for two weeks on healthy grapevines in both locations. ^d^ Summation of vines testing positive for GRBV via PCR over the total vines tested at each geographical location. ^e^ Number of vines for which leaf samples tested positive for GRBV by PCR over the total number of vines exposed to viruliferous *S. festinus*; n/a: not applicable. ^f^ Summation of vines testing positive for GRBV via PCR over the total vines tested based on sleeve configuration. ^g^ Twenty *S. festinus* were allowed to feed on the entire length of a shoot. ^h^ Ten *S. festinus* were allowed to feed on a restricted area of a shoot. ^i^ Two *S. festinus* were allowed to feed on a single leaf. ^j^ Summation of vines testing positive for GRBV via PCR over the total vines tested for each year that the experiments were conducted in.

**Table 2:** Number of girdles and red leaves observed on shoots exposed to viruliferous *Spissistilus festinus* during the 2021 growing season in New York using three different sleeve configurations.

Sleeve Configuration	Vine Number	Girdles ^a^	Red Leaves ^b^	GRBV ^c^
Lollipop ^d^	1	3	0	-
	2	3	0	-
	3	2	0	-
	4	1	0	-
	5	2	0	-
Peppermint candy ^e^	1	15	1	-
	2	23	1	-
	3	10	3	-
	4	19	2	-
	5	16	2	-
	6	16	2	-
	7	9	0	-
	8	12	2	-
	9	14	5	-
	10	13	3	-
	11	10	2	-
	12 *	15	3	-
Tootsie roll ^f^	1	17	5	-
	2	30	4	-
	3	21	6	-
	4	20	9	-
	5 *	25	4	-

^a^ Total count of girdles observed within the sleeves of inoculated shoots (peppermint candy and tootsie roll) or leaves (lollipop). ^b^ Total number of red leaves observed within the sleeves of inoculated shoots (peppermint candy and tootsie roll) or leaves (lollipop) through the remainder of the growing season. ^c^ Results of multiplex PCR testing of inoculated tissue for grapevine red blotch virus (GRBV). ^d^ Two *S. festinus* fed on a single leaf. ^e^ Ten *S. festinus* fed on a restricted area of a shoot. ^f^ Twenty *S. festinus* fed on the entire length of a shoot. * Grapevines exposed to aviruliferous *S. festinus*.

## Data Availability

Raw data will be made available upon request.
